# Understanding the Psychosocial Correlates of the Intention to Use Condoms among Young Men in KwaZulu-Natal, South Africa

**DOI:** 10.3390/ijerph14040339

**Published:** 2017-03-23

**Authors:** Thabang Manyaapelo, Anam Nyembezi, Robert A. C. Ruiter, Bart van den Borne, Sibusiso Sifunda, Priscilla Reddy

**Affiliations:** 1 Human Sciences Research Council, Population Health, Health Systems and Innovation, Private Bag X41, Pretoria 0001, South Africa; 2 Human Sciences Research Council, Population Health, Health Systems and Innovation, Private Bag X9182, Cape Town 8000, South Africa; anyembezi@hsrc.ac.za (A.N.); preddy@hsrc.ac.za (P.R.); 3 Department of Work & Social Psychology, Maastricht University, P.O. Box 616, 6200 Maastricht, The Netherlands; r.ruiter@maastrichtuniversity.nl; 4 Department of Health Education & Health Promotion, Maastricht University, P.O. Box 616, 6200 Maastricht, The Netherlands; b.vdborne@maastrichtuniversity.nl; 5 Human Sciences Research Council, HIV/AIDS, STIs and TB, Private Bag X41, Pretoria 0001, South Africa; ssifunda@hsrc.ac.za

**Keywords:** condom use, HIV/AIDS, risky sex, substance use, African men, theory of planned behaviour, responsible manhood

## Abstract

South Africa leads the world with the number of people infected with HIV. Even with all attempts that have been made to curb HIV, it is still evident that new infections are on the rise. Condom use remains one of the best tools against this challenge yet a small number of sexually active men use them. This study investigates the psychosocial correlates of the intention to use condoms among young men in KwaZulu-Natal province. Using the Theory of Planned Behaviour as a framework, hierarchical linear regression models were used to determine the unique contribution of the study measures in explaining the overall variance of intention to consistently use condoms. Subjective norms and perceived behavioural control towards consistent condom use explained 46% of the variance in the intention to use a condom, suggesting that health behaviour interventions should focus on targeting the normative beliefs as well as control beliefs of the target population. Furthermore, subjective norms and intentions towards reducing alcohol and marijuana use explained an additional 7% to the final model in intentions to condom use, implying that substance use and condom usage may influence each other. No significant contributions were found for beliefs underlying cultural aspects of responsible manhood.

## 1. Introduction

South Africa is battling to control incidents of new HIV infections. The latest South African National HIV Prevalence, Incidence and Behaviour Survey reported that approximately 6.4 million South Africans were living with HIV, with the number of new annual recent infections reported at about 460,000 and about 4 million people on anti-retroviral therapy (ART) [1,2]. In 2015, the number of people living with HIV had risen to 7 million, with KwaZulu-Natal (KZN) province reported as having the highest HIV prevalence at 17.4% and the most affected group being people between 15 and 49 years of age [1,3].

Several HIV prevention strategies have been used to curb the HIV epidemic in KZN, in line with national policies and programs, among which is voluntary medical male circumcision (VMMC) coupled with HIV Counseling and Testing (HCT). The World Health Organization (WHO) recommended VMMC as an additional HIV prevention option [4]. The WHO policy position was influenced by the three randomised controlled trial (RCT) studies conducted in Uganda, Kenya and South Africa, which found that VMMC reduced HIV infections in men by between 55% and 61% [5,6,7]. The high HIV prevalence prompted the national government to deploy more resources towards VMMC in KZN, where between 2010 and 2014 the province reported more VMMC than the other eight provinces [8].

In as much as VMMC is seen to play a role towards HIV prevention in KZN communities, consistent condom use remains one of the most effective prevention tools against HIV transmission [9]. It is for this reason that we have to understand as much as we can about the determinants of condom use in the various populations where the scourge of HIV remains quite high. Given the high prevalence of HIV in South Africa, condom use becomes pivotal due to the fact that more people living with HIV are now enrolled in ART. Previous studies have reported that the status of being on ART, of having undergone VMMC and of knowing one’s HIV status are associated with an increased likelihood to engage in risky sex, which is often attributed to men refusing to use condoms [10,11,12,13]. Therefore, it is very critical that both infected and uninfected people should be encouraged equally to use condoms consistently and correctly. It should be highlighted that condom use in this study only refers to the latex male condom typically used for vaginal or anal intercourse.

This study utilised the Theory of Planned Behaviour (TPB) as the basis from which to investigate the intentions to use condoms among a group of sexually active young men. The Theory of Planned Behaviour (for a recent re-formulation see Fishbein and Ajzen [14]) posits that behavioural intention is determined by the independent variables attitude, subjective norm, and perceived behavioural control. Attitude represents a person’s favourable or unfavourable evaluation of the anticipated outcomes of the behaviour, and is the product of the individual’s behavioural beliefs regarding the likely outcomes of performing the behaviour. Subjective norms are a product of the normative expectations of other meaningful people towards a particular behaviour and the motivation to comply with the opinions of those people. Lastly, perceived behavioural control is determined by an individual’s beliefs whether there are barriers to their control over the behaviour and the perceived power the individual feels to remove these barriers [15].

Many studies have investigated the intention to use condoms using the Theory of Planned Behaviour (TPB) as a theoretical framework [16,17,18,19,20,21]. These studies vary in their prediction of the TPB construct most influential in predicting the intention to use condoms. Some studies have shown subjective norms to be the strongest cognitive predictor of intention to use condoms [22,23,24], others emphasised attitudes and perceived behavioural control as strongest [25,26], and there are those that showed the predictive power of attitude, subjective norms and perceived behavioural control in varying combinations [27,28]. These studies highlight most importantly that, when researchers are designing behavioural health interventions, it is essential that they take the context of the targeted populations concerned into account. To this end some researchers have called on the Theory of Planned Behaviour proponents to be cognizant of cultural contexts [29,30]. In response to this, the TPB has been shown to have good predictive capabilities beyond a Western context where it was first developed [31], and TPB, like other theories, describes processes that can be generalized over groups and across cultures. That is, for example, the construct of behavioural beliefs influences the general evaluation of a behaviour that we call attitude, which in turn determines the behavioural response, but the content of the beliefs may vary across cultures [32].

In South Africa, more specifically, a few studies have used the Theory of Planned Behaviour as a framework to research gender power imbalances in the intention to use condoms [33], the role of individual and group factors in intentions to use condoms [34], adolescent condom use behaviour [25,35,36], the influence intention has on behaviour among university students [37], and condom use motivation in traditional male circumcision initiates [38]. These studies also vary in their prediction of the most influential cognitive constructs, and some also show a clear gender difference in their predictability where both sexes were sampled. However, there is a paucity of evidence with regards to the determinants of condom usage studies for South African men. Young men between the ages of 18 and 35 are particularly at a high risk of HIV infection. Given the gender imbalances in HIV transmission, it is expected that developing culturally sensitive interventions targeting young men will help increase safer sexual practices [38]. A focus on young men can also help impact the gender imbalances reported by some studies that, in heterosexual relationships, women do not use condoms mainly because of their sexual partner’s refusal to use them [10,11]. Studies examining men who have sex with men report that the reasons for not using condoms are pressure from the sexual partner as well as distrust [39]. What is also evident is that condom problems and condom failures were seen among this group as being normal.

In order to identify a broader range of factors that serve as possible determinants of condom use, this study extended the measures related to condom use by including measures that relate to substance use and the concept of responsible manhood. Previous research has found that excessive alcohol intake (>14 drinks/week for men and >7 drinks/week for women) is associated with an increased sexually transmitted disease acquisition [40]. Alcohol use and marijuana use have also been shown to adversely affect higher order cognitive processes often classified as executive control functions, including time estimation, attention, planning, behavioural flexibility, decision-making, and inhibitory control [41,42,43,44]. Some of these basic cognitive functions play an important role in sexual decision-making.

We introduced the concept of responsible manhood because part of the study population is drawn from traditional communities in KZN. Within these communities there are traditional notions of responsible manhood, which underpin behaviour. Previous studies in their attempt to define manhood have done so in terms of the roles that males play in society [45,46,47], which is mostly defined against the dominant background of Western culture. In this context of Western culture, the stereotypical masculine traits of physical strength, dominance, control, emotional restraint and assertiveness together with being white and heterosexual are associated with contemporary hegemonic masculinity [48,49,50]. Manhood in the African context is a lifelong process of a systematic socialization of the boy child, wherein the mores of the community this young man is brought up in are entrenched through marked initiation rites of passage. Some of these life stages include male traditional circumcision [51] and acquiring resources for a future marriage, which is understood to be a sign of maturity and responsibility, while acts of irresponsibility such as impregnating girls are vehemently discouraged with payable fines [52]. This new construct of responsible manhood is derived from the traditional concepts of supportive male roles together with discouraging, hurtful behaviours. It is hypothesised that this construct will help explain some of the determinants of condom use among these young men.

The psychosocial and behavioural complexities in South Africa are characterized by a population of people living with HIV, of people on ART and of people who remain unaware of their HIV status. This intricate landscape calls for rigorous theory-based inquiry into the determinants of condom use. This paper will attempt to add to the scant literature on the psychosocial determinants of condom use in African men, and further attempt to answer the call for health behavioural interventions to have foundations in sound health behaviour theory [53,54,55].

## 2. Materials and Methods

### 2.1. Study Design

This article reports on the baseline data which forms part of a larger dataset collected in the development and testing of a Health Behaviour Intervention targeted at young men in the province of KZN. The intervention focused on behavioural key points that promote consistent condom use, promote the reduction of sexual partners, encourage testing for HIV, discourage the use of alcohol and drugs, and lastly encourage the young men to play more supportive positive male roles in their respective communities. The study received full ethical clearance from the South African Medical Association Research Ethics Committee (SAMAREC- Protocol MRC 1-09), and additionally the research team also sought and received permission from the local municipal offices and the traditional leadership in the area concerned. Participants gave written informed consent to participate in the study.

### 2.2. Participants and Study Setting

The study was conducted in the province of KwaZulu-Natal on the North-Eastern coast of South Africa. It is the second most populated province at 10.8 million people with 86.1% being African, 7.9% Indian/Asian, 4.6% White, and 1.4% Coloured [56,57]. The sample comprised of males between the ages of 18 and 35 who were mainly isiZulu speaking, resided in the area and indicated availability for a follow-up at 6 months post intervention. The research participants were recruited from multiple community sites such as schools, churches, and community organizations. Participation in the study was on a voluntary basis to all participants who met the inclusion criteria and were able to come and take part during the times allocated. Researchers provided transport (where necessary) and also made provision for refreshments to the participants.

The recruitment drive included a well-publicised initiative of talks about the study aims at community meetings, local churches and sports tournaments organised specifically for this purpose. The research team was also hosted at a local community radio station to field questions from the community. This recruitment drive continued for nearly 12 months before commencing the study. Site A is a peri-urban locality roughly 30 km from Durban with a majority African population, while Site B is rural and approximately 250 km from Durban, also with a majority African population. A total of 575 young men completed the baseline questionnaire, of whom 350 responded “yes” to “having had one or more sexual partners in past 6 months”. A sexual partner was defined as any person with whom the participant had either vaginal or anal sex.

### 2.3. Study Instruments

Data was collected through a facilitator-administered questionnaire. This questionnaire was adapted from a previous study among male prison inmates in KwaZulu-Natal and Mpumalanga provinces [58]. Additionally, the content for the questionnaire was derived from a literature review on the topic as well as focus group interviews among the study group. The name of this adapted questionnaire was the same as that of the main study: Ubudoda Abukhulelwa Responsible Manhood: Towards the Development of Culturally Tailored and Contextually Sensitive Life Skills Programs for Heterosexual Men in South Africa. The questionnaire was divided into three sections, where the first measured the demographic profile of the population in terms of age, level of education, level of income, and whether participants were involved in sexual relationships or not. The second section examined the participants’ sexual risk behaviour in terms of the number of sexual partners and current substance use behaviours concerning alcohol and marijuana use and their frequency. The last section focused on the psychosocial measures constructed using the Theory of Planned Behaviour, where each behaviour was measured for attitudes, subjective norms, perceived behavioural control, and intentions. The questionnaire was developed in English and translated into isiZulu, then it was translated back into English to ensure construct and face validity. The research assistants together with the project managers, who came from the same background as the research participants, were responsible for the translation process. The translations were all done in the form of a workshop with all the research assistants, project managers, and some of the co-authors (Thabang Manyaapelo, Sibusiso Sifunda, Anam Nyembezi) in attendance. Consensus was reached for the correct use of language for all the research tools.

### 2.4. Measures and Scale Construction

#### 2.4.1. HIV Knowledge

Ten single items measured knowledge of HIV using: 1 = True, 2 = False, and 3 = I don’t know response options. (Example: The HIV virus can be passed from a pregnant mother, if she is infected with HIV, to her unborn child. The responses were dichotomised as 1 = True and 0 = False or I don’t know).

#### 2.4.2. Condom Knowledge

Three single items measured knowledge about condoms using a scale of 1 to 5: 1 = fully disagree, 2 = disagree, 3 = unsure, 4 = agree, and 5 = fully agree. (Example: Condoms work well to prevent the spread of HIV. The responses were dichotomised as 1 = Fully agree or Agree, 0 = Unsure, Disagree or Fully Disagree).

#### 2.4.3. Multiple Partners

Risky sexual behaviour was measured by asking the number of sexual partners the participant had engaged in sex with in the past six months. A four-point scale was used with answering options of: 0 = not sexually active; 1 = 1 sexual partner; 2 = between 2 and 5 sexual partners, 3 = between 6 and 10 sexual partners, and 4 = 10 or more sexual partners.

#### 2.4.4. Alcohol and Marijuana Use

Two single items assessed the frequency of alcohol and marijuana use in the past six months, respectively, using a five-point scale with options of: 1 = never (0 days), 2 = rarely (1 to 2 days), 3 = sometimes (3 to 9 days), 4 = often (10 to 19 days), and 5 = very often (20 days or more).

#### 2.4.5. Psychosocial Correlates

The Theory of Planned Behaviour variables (attitude, subjective norm and intention) toward condom use for every sexual encounter consistently in the next three months were measured using a scale of 1 to 5 with answering options of: 1 = strongly/fully disagree, 2 = disagree, 3 = unsure, 4 = agree, and 5 = strongly/fully agree for attitude, subjective norm and intention, while perceived behavioural control was measured using a scale of 1 to 5 with options of: 1 = very confident; 2 = confident, 3 = unsure; 4 = not confident, and 5 = not confident at all. Table 1 provides an overview of the psychosocial correlates that were measured, including the number of items, sample items, minimum and maximum score, and Cronbach’s Alpha (three or more items) or Pearson’s r (two items) as a measure of the internal consistency of grouped items.

### 2.5. Analysis

Statistical analysis was done using SPSS Version 23 (Statistical Product and Service Solutions, IBM, New York, NY, USA). Bivariate correlations analysis was used to assess associations between study measures. Hierarchical linear regression models were then used to determine the unique contribution the study measures made to explaining the overall variance in the intention to use a condom consistently with every sexual encounter. The regression was done in a five-step process starting with the more proximal predictors and ending with the more distal predictors. In step 1, the outcome variable is tested against the most proximal predictors of attitude, subjective norm and perceived behavioural control towards the behaviour of interest (using a condom consistently with every sexual encounter). In step 2, attitudes, subjective norm, perceived behavioural control, and intention towards reducing alcohol and marijuana used were added. Step 3 added the HIV and condom knowledge. Step 4 added attitude, subjective norm, perceived behavioural control, and intention towards responsible manhood. Finally, the demographic variables (age, level of education) and behavioural variables (past substance use and sexual behaviour) were added in step 5.

## 3. Results

### 3.1. Socio-Demographic Profile of the Participants

A total of 350 sexually active young men were included in this analysis. The ages ranged from 18 to 35, with the majority (64.9%) between the ages of 18 and 20 (see Table 2). The level of education for this sample varied from primary education to tertiary education, with 40% (*n* = 143) of the sample having a grade 12 or higher qualification. Almost all participants reported being unemployed (96.5%). Most participants lived with at least one parent or with a relative.

### 3.2. Sexual Behaviours and Substance Use

About 73% reported having multiple concurrent sexual partnerships. There was reasonably adequate knowledge regarding use of condoms, with 78.8% and 81.3% believing that “condoms can prevent the spread of HIV and prevent pregnancies”, respectively. With regards to general perception towards using condoms, 41.4% believe that condoms “take the fun out of sex” while 30.1% think that using a condom “shows that you do not trust your partner”. Just over three quarters (78.3%) reported having used alcohol in their lifetime and 38.9% reported having used marijuana. A total of 36.3% of the participants used both alcohol and marijuana.

### 3.3. Predictors of Intention to Use a Condom Consistently with Every Sexual Encounter

Table 3 presents the correlations as well as the results of the hierarchical stepwise analysis including the predictors of intention to use a condom consistently with every sexual encounter.

The intention to use a condom consistently with every sexual encounter in the next three months showed strong positive correlations with subjective norms (*r* = 0.61) to use a condom consistently, and the subjective norm (*r* = 0.51) and intention (*r* = 0.52) to reduce alcohol and marijuana use. A moderate positive correlation was found for perceived behavioural control to use a condom consistently with every sexual encounter (*r* = 0.23). Weak positive correlations were found for attitude towards consistent condom use, attitude and perceived behavioural control towards reducing alcohol and marijuana use, and attitude, perceived behavioural control and intention towards behaving as a responsible man. Condom knowledge and HIV knowledge also showed significant but weak positive correlations.

In the first step of the multiple regression analysis, attitude, subjective norm, and perceived behavioural control explained 46% of the variance in the intention to use a condom consistently with every sexual encounter, showing significant contributions of subjective norm and perceived behavioural control. The second step explained an additional 7% of variance (*p* ≤ 0.001), with additional significant contributions of subjective norm and intention towards reducing alcohol and marijuana use. The third step added the condom and HIV knowledge variables, which did not add any additional variance. The fourth step introduced the responsible manhood variables, which did not add any additional variance to our model, but removed the significant contribution of perceived behavioural control towards using a condom consistently with every sexual encounter. Finally, in the fifth step, adding demographic variables and past alcohol and marijuana use also explained no additional variance. The final model explained 52% of the variance in the intention to use a condom consistently with every sexual encounter with unique significant contributions of the subjective norm toward consistent condom use as well as the subjective norm and intention to reduce alcohol and marijuana use.

## 4. Discussion

Condom usage at the most recent sexual encounter, as reported in the 2012 South African National HIV Prevalence, Incidence and Behaviour Survey, dropped from 45.1% to 36.2% between 2008 and 2012 for both men and women across all age groups [1]. Various reasons have been given for this, the first being that condom usage may have initially been overestimated, secondly that prevention messages promoting condoms were no longer receiving the attention they used to get, and lastly that perhaps because of the widely available ART, people now engage in risky sexual behaviour as they deem protection unnecessary [1]. The need to understand the psychosocial determinants that could have contributed to this condom use decline is more serious than previously thought, particularly among communities who are at a high risk of HIV infection. Our sample is at an increased risk of STIs, including HIV infection, because 73% of the participants reported having multiple sexual partnerships, which makes them highly susceptible to HIV infection spread through unprotected sex [1].

The objective of this paper was to investigate the determinants of the intention to use condoms among young men living in KZN, using the Theory of Planned Behaviour as the guiding framework. We found that the intention to use a condom consistently with every sexual encounter showed strong correlations with the subjective norm to use a condom consistently. Most notably we found that the proximal TPB constructs of subjective norms and perceived behavioural control explained a total of 46% of the variance in the intention to use a condom consistently with every sexual encounter. This finding is comparable to other studies [33,34,36,59] in African countries, which have also shown subjective norms to be a stronger cognition predictor of intention to use condoms when it is compared to other proximal cognition constructs, namely attitudes and perceived behavioural control. The total variance in intention to use condoms explained in these other studies ranges between 22% and 67%, however it has been recently reported that in general the predictive value for TPB constructs in sub-Saharan Africa is on average less than that of North American and European studies [36].

With subjective norms seen as prominent in the current study, this suggests that young men in the KZN community are strongly influenced by what significant people in their lives think about their behaviours. The young men have a strong motivation to comply with the opinions of these people. This finding supports the assertion that decision-making in indigenous African communities is more communal, collaborative, and less individualistic [29,34]. The structure of living arrangements in South African communities, particularly the ones surveyed in the present study, is such that the nuclear family as often seen in the Western world is rare. Less than half of the participants live with at least one parent (47.8%). The majority of these participants live in small dwellings with their siblings and extended family members.

Furthermore, our findings suggest that the young men have a strong belief in their own ability to use condoms consistently with every sexual encounter, this being supported by the significance of perceived behavioural control as a predictor of the intention to use condoms, which was similarly found in other studies [60,61]. The reasons for this strong belief may be influenced by a number of factors. It has been shown that condom use intention does not automatically translate into actual behaviour without certain important preparatory behaviours like buying and carrying the condom, being able to negotiate its use with the sexual partner, and lastly having the skills to use it correctly [62]. The strong belief in their own ability to use condoms exhibited by the young men under study could be attributed to any one or more of these considerations. With regards to just access to condoms alone, in South Africa, government health facilities provide free condoms. Gender inequality and intimate partner violence have been shown to be important factors when considering condom usage in sexual relationships [63]. These gender dynamics may play a role in making young men more confident in their power to use condoms in the future. Previous research has shown how existing social norms and social power are correlated, and that individuals with social power tend to influence social norms [19]. The power relations in condom use negotiation reveal that women mainly attributed the lack of condom usage to the fact that their sexual partners refused to use it [10], while other research details multiple tactics men were found to employ in their attempt to avoid condom use [11], therefore the confidence seen in the young men under study could be due to their knowledge that they hold the power to dictate condom usage in their respective sexual relationships. The findings by Eisele et al. [10] are however contradictory to Bryan et al. [35], who had earlier found that in heterosexual relationships, it was mostly girls who reported significantly higher control over sexual encounters, therefore Bryan et al. concluded that it was girls who mainly decide whether a sexual activity will occur or not. It is interesting that both these studies with contradictory findings were conducted in South Africa, which should motivate further research into the determinants of condom use negotiation in these communities. Research into the determinants of condom use should also be cognizant of the evidence in support of the correct usage of condoms, whereby condom breakage, slippage and the use of condom-compatible lubrication are taken into account [39,64,65].

When substance use variables were included into the model, we found that the intention to use a condom consistently with every sexual encounter showed strong correlations with the subjective norms and intention to reduce alcohol and marijuana use. In the regression both subjective norms and intentions to reduce alcohol and marijuana use were shown to explain a further 7% of the variance in the intention to use a condom consistently with every sexual encounter. The young men who had positive intentions towards reducing risky behaviour for alcohol and marijuana use also intended to use condoms with every sexual encounter. This finding is comparable to a recent study [66] which reported similarly that surveyed participants who had strong intentions to reduce harmful substance use were also more likely to avoid engaging in risky sexual encounters. Again, here we see how meaningful others (i.e., the people who are in the participant's immediate environment and whose opinions are regarded as important in decision-making) play an important role in their intention towards more positive behaviour choices. For example, customs dictate that a father’s brother or a mother’s brother be afforded the same respect as the father. These older men can exercise considerable influence on our participants, especially in instances where fathers work far from the home, which is commonplace in South Africa.

Condom and HIV knowledge variables showed significant but weak correlations with the intention to use a condom consistently with every sexual encounter, and were not shown to be significant for the regression. The more knowledge the participants have of condoms and HIV, the more likely they were to use a condom. In previous studies, it has been shown that knowledge does not necessarily have an impact on behaviour. More recently it has been reported that it is actually the quality of the knowledge and not that amount that is important in determining condom usage for both males and females [67].

Past behaviour related to substance use did not yield any significant correlations and did not explain any additional variance. Previous studies show that the type of behaviour (whether habitual or not) as well as the context play an important role in predicting future behaviour [18,68]. Simply put, it means that the more a person engages in a specific behaviour in a specific context, the more the initiation and control processes of that behaviour become automatic. In situations where there are unstable contexts such as in condom use, it becomes difficult to maintain this automatic process. This lack of significance is further attributed to the theoretical notion of TPB that the effect of past behaviour is mediated by more proximal cognitions [69]. Typically, the relationship between behaviour, such as condom use and alcohol or marijuana use, is present in that moment when the individual is intoxicated so it is expected that past substance use will not predict future intentions to use condoms.

Level of education did not show any significant correlation with the intention to consistently use a condom and did not add any additional variance. In previous research, condom use was associated with higher levels of education [70]. This lack of significance could also be due to the variable being mediated by more proximal cognitions.

The newly constructed responsible manhood variables showed significant but weak correlations for attitudes, perceived behavioural control and intentions towards being a responsible man, however, these variables didn’t explain any additional variance. Although the addition of these responsible manhood variables did not explain any additional variance, it is interesting to note that they cancelled the significance of perceived behavioural control to use a condom consistently in the final model, implying that notions of responsible manhood and control beliefs could be more intricately linked. Understanding the role of the concept of responsible manhood definitely warrants more investigations.

Our findings suggest that interventions to use condoms should target both the normative beliefs and control beliefs towards using condoms consistently, and also target the psychosocial correlates towards the reduction of alcohol and marijuana use. This can be achieved, for example, by showing positive peer support for risk prevention and by building personal resistance against social pressures for risk-taking behaviour, and by enabling physical as well as social environments that facilitate condom use. Facilitating free access to condoms at drinking establishments is one way. Another way could be to have prominent people in targeted communities speak publicly about using condoms and exercising safe sexual practices.

## 5. Limitations

A possible limitation of this study is the responsible manhood concept, which should be investigated and developed further. A potential reason why this study was unable to show strong correlations with the responsible manhood constructs may be because the constructs and variables were recently developed and did not thoroughly test what had been intended. As this concept is still in the development stages, the constructs and variables should be further developed and validated for future studies. The results showed that about a third of men in the study engage in substance use. These figures may well be inflated due to the large number of unemployed men sampled. This bias could have inadvertently been as a result of the inclusion criteria, which restricted availability of the potential participants to working hours during the week (08:00 to 17:00, Mondays to Fridays).

## 6. Conclusions 

The findings in this study show that subjective norms and perceived behavioural control are important cognitive constructs in the prediction of intention to use condoms consistently among young men in KZN, a province in South Africa. Our research results therefore imply that health education interventions should focus on changing the normative beliefs as well as control beliefs of the target population, but also encourage substance use reduction interventions since substance use is negatively associated with condom use.

## Figures and Tables

**Table 1:** Overview of scale measures with examples.

Measures and Example Items	Number of Items	Min Score	Max Score	Cronbach’s Alpha (α)/Pearson’s r
**Attitudes towards using a condom consistently for every sexual encounter in the next three months** - Using a condom consistently for every sexual encounter in the next three months is something that is good	5	1	5	0.81
**Subjective Norms towards using a condom consistently for every sexual encounter in the next three months** - Most people who are important to me think that using a condom for every sexual encounter consistently in the next three months is a good thing	5	1	5	0.88
**Perceived Behavioral Control towards using a condom consistently for every sexual encounter in the next three months** - For me to use a condom consistently for every sexual encounter in the next three months is possible	3	1	5	0.70
**Intentions towards using a condom consistently for every sexual encounter in the next three months** - I intend to use a condom consistently for every sexual encounter in the next three months	6	1	5	0.92
**Attitudes towards reducing overall alcohol and drug intake** - Reducing overall drug and alcohol intake to only one day a week in the next three months is something that is wise	6	1	5	0.84
**Subjective Norms towards reducing overall alcohol and drug intake** - Most people who are important to me think that reducing overall drug and alcohol intake to only one day a week in the next three months is a good thing	5	1	5	0.84
**Perceived Behavioral Control towards reducing overall alcohol and drug intake** - For me to reduce overall drug and alcohol intake to only one day a week in the next three months is possible	3	1	5	0.66
**Intentions towards reducing overall alcohol and drug intake** - I intend to reduce overall drug and alcohol intake to only one day a week in the next three months	7	1	5	0.93
**Attitudes towards behaving as a responsible man** - A responsible man is someone who has to discipline his wife/partner when necessary using physical force	2	1	5	0.59
**Subjective norms towards behaving as a responsible man** - Most of your community members think that a responsible man is someone who has to discipline his wife/partner when necessary using physical force.	2	1	5	0.73
**Perceived Behavioral Control towards behaving as a responsible man** - How confident are you that you will be able to look after your partner’s wellbeing?	2	1	5	0.42
**Intentions towards behaving as a responsible man** - I intend to discipline my wife/partner when necessary using physical force in the next three months	1	1	5	-

**Table 2:** Socio-demographic profile of the participants.

Characteristic	Frequency	Percentage
Age		
18–20	226	64.90%
21–25	85	24.40%
26–30	30	8.60%
31–35	7	2.00%
Levels of education		
Primary	1	0.30%
Standard 6	33	9.90%
Standard 8	156	46.80%
Matric	100	30.00%
Tertiary (Technikon)	25	7.50%
Tertiary (University)	18	5.40%
Participants not employed	335	96.5%
Participants living on their own	34	10.5%
Participants living with at least one parent	108	47.8%
Participants living with a relative	42	18.6%

**Table 3:** A stepwise hierarchical regression testing the constant, intention to use a condom consistently with every sexual encounter against the predictors, substance use, knowledge, responsible manhood, demographic and past behaviour variables.

Model	*r*	Step 1		Step 2		Step 3		Step 4		Step 5	
		b	β	b	β	b	β	b	β	b	β
**Use condoms consistently with every sexual encounter **											
attitude	0.113 *	0.02	0.01	−0.03	−0.02	−0.04	−0.02	−0.03	−0.02	−0.03	−0.02
subjective norm	0.619 **	0.61	0.65 ***	0.49	0.52 ***	0.49	0.52 ***	0.48	0.51 ***	0.48	0.52 ***
perceived behavioural control	0.230 **	0.13	0.10 ***	0.13	0.10 ***	0.13	0.10 ***	0.12	0.09	0.11	0.08
**Reduce alcohol and drug use**											
attitude	0.137 *			−0.04	−0.03	−0.04	−0.03	−0.03	−0.03	−0.04	−0.03
subjective norm	0.517 **			0.14	0.13 ***	0.14	0.12 ***	0.14	0.12 ***	0.15	−0.13 ***
perceived behavioural control	0.135 *			−0.01	−0.00	−0.02	−0.02	−0.03	−0.02	−0.03	−0.02
intention	0.528 **			0.23	0.22 ***	0.23	0.21 ***	0.23	0.21 ***	0.23	0.21 ***
**Knowledge of HIV and condoms**											
Condom knowledge	0.141 **					0.02	0.00	0.01	0.00	0.01	0.00
HIV knowledge	0.143 **					0.38	0.06	0.35	0.05	0.31	0.05
**Responsible Manhood**											
attitude	0.105 *							0.01	0.02	0.01	0.02
subjective norm	0.017							0	0	−0.00	−0.00
perceived behavioural control	0.161 **							0.02	0.03	0.02	0.03
intention	0.122 *							−0.01	−0.01	−0.01	−0.01
**Demographic **											
age	0.03									−0.01	−0.00
level of education	0.092									0.07	0.06
relationship status	−0.044									0.13	0.06
**Past behaviour **											
marijuana use	0									−0.00	−0.00
alcohol use	−0.022									−0.04	−0.03
number of sexual partners	−0.043									0.03	0.02
**Constant**		1.14		0.52		0.35		0.20		−0.18	
**F-change**		87.7 ***		11.1 ***		1.1		0.2		1.0	
**Adjusted R Square**		0.46		0.53		0.53		0.52		0.52	

Note for regression *** *p* < 0.05 and for correlations ** *p* < 0.01, * *p* < 0.05; b = unstandardized Beta, β = standardized Beta, *n* = 350.
